# Treatment of Abortion

**Published:** 1896-08

**Authors:** J. W. Long

**Affiliations:** Richmond, Va., Professor of Diseases of Women and Children, Medical College of Virginia


### TREATMENT OF ABORTION.*

By J. W. LONG, M.D., RICHMOND, VA.,

Professor of Diseases of Women and Children, Medical College of Virginia.

A consideration of this subject involves many interesting questions. Of these I will discuss but a few, and briefly.

1. *When is abortion inevitable?*

This question settled, the course to be pursued will be clear. Uterine pains and hemorrhages have been considered as sure evidences that abortion will occur. That this is not always true is easily proven. I recall one case that bled from the first till after the third month (with recurring pains) that went to term. Scanzoni reports a case in which occurred profuse hemorrhages on the third month, and in spite of ergot, the tampon, use of the uterine sound, and an intrauterine injection of solution of perchloride of iron, the case went on to term. Noble† was called to a case that resisted the repeated applications of pure carbolic acid to the endrometrium. Better indications that abortion is inevitable are, dilatation of the os and descent of the ovum. Escape of the liquor amnii, or septic infection, may be considered positive proof that the expulsion of the fetus is inevitable.

2. *Treatment when abortion is not inevitable.*

Rest, quiet, and opium are the sovereign remedies. A friend of mine kept a patient under the influence of opium the entire last

*Read before the Richmond Academy of Medicine and Surgery, February 25, 1896.

†A Consideration of Certain Doubtful Points in the Management of Abortion. Dr. Chas. P. Noble.

seven months of her pregnancy. Pains would begin whenever the drug was discontinued, nor did she become an opium habitué.

3. *Inevitable abortion.*

The plain indication here is to empty the uterus, which, according to the classic maxim, is not complete until the fetus, placenta, and all clots are removed. Many times nature herself is able to do this, and only when she fails are we to interfere.

By far the best instrument for removing the products of conception is the finger, aided by the other hand depressing and steadying the fundus from above. In those cases where the cervix is not patulous, it becomes necessary to dilate the cervix with a steel dilator. Then the cavity is to be explored by the finger or curette, followed by a copious irrigation and a pack of iodoform gauze.

Sometimes one is in doubt as to whether the uterus is empty. Especially is this true of abortions in the third and fourths months. The ovum passed intact is evidence that the uterus is empty. A patulous cervix and continued bleeding indicate that something yet remains in the uterus.

4. *Septic abortion.*

In cases of this kind, delay is inexcusable. Many deaths may be put down to the credit of the let-alone, waiting, non-interference policy. The products of conception broken up and infected, as in criminal abortion, present the ideal condition for producing general sepsis. Not only should the products of conception be removed, but also the infected maternal membranes. The gaping lymphatics and open fallopian tubes will continue to spread the infection as long as any remains in the uterus. Therefore the necessity for a thorough cleansing. In most instances this can only be done by the curette. In more advanced cases the finger is a safe guide, because sentient. Thorough irrigation and packing are also essential.

I have not stated that all operative or explorative procedures in the uterus must be done under strict antiseptic precautions, as this goes without saying, and is hardly pertinent to the question under consideration.

I doubt that it is justifiable to curette more than once in these cases; indeed, I question whether or not uterine irrigations should be continued longer than one or two days, and it is quite probable that in these cases where the local inflammation involves the peri-uterine to a marked degree, the manipulations necessary to intra-uterine irrigation do more harm than good.

5. *When shall further operative procedures be instituted?*

*Hysterectomy* for puerperal sepsis is one of the burning questions of the day. The advocates for it have not yet made out their case, but have established just claims to be heard. The most that can be said, at this writing, for the operation is that hysterectomy is indicated in those cases of sepsis where thorough irrigation and curettage have failed to modify or abate the general sepsis and *the local septic inflammation is confined to the uterus.* I emphasize the words in italics, for thereupon will depend the result. Two such cases were reported by Dr. Cartledge, of Louisville, at the Washington meeting of the Southern Surgical and Gynecological Association, the uterus in each case being honeycombed with septic abscesses.

*Vaginal incision and drainage* is an operation of great value in just those cases where hysterectomy is contraindicated.

I have described the indications and technique in a paper read by title before the Southern Surgical and Gynecological Association, at the Washington meeting in November, 1895. By this method the sodden, septic pelvic tissues are drained of the sepsis, and the general system is also relieved.

---

I AM not an agnostic in medicine, though I do not believe everything that medical quidnuncs tell me. My repertory of drugs is very small, viz.: mercury, arsenic, iron, tartarized antimony, sulphate of magnesia, iodine, opium, quinine, chloroform, chloral, nux vomica, the bromides and synthetic remedies. These are my whole armamentarium, although I can easily manage with less, provided I have the assistance of cold water and the *vis medicatrix naturæ.* All the rest is "leather and prunella."—Dr. JOHN MORRIS, in *Maryland Med. Journal.*

